# Tightly-Coupled Stereo Visual-Inertial Navigation Using Point and Line Features

**DOI:** 10.3390/s150612816

**Published:** 2015-06-01

**Authors:** Xianglong Kong, Wenqi Wu, Lilian Zhang, Yujie Wang

**Affiliations:** College of Mechatronics and Automation, National University of Defense Technology, Changsha 410073, China; E-Mails: kongxianglong51@gmail.com (X.K.); lilian-zhang@hotmail.com (L.Z.); yjwang@nudt.edu.cn (Y.W.)

**Keywords:** vision-aided inertial navigation, point and line features, trifocal geometry, tightly-coupled

## Abstract

This paper presents a novel approach for estimating the ego-motion of a vehicle in dynamic and unknown environments using tightly-coupled inertial and visual sensors. To improve the accuracy and robustness, we exploit the combination of point and line features to aid navigation. The mathematical framework is based on trifocal geometry among image triplets, which is simple and unified for point and line features. For the fusion algorithm design, we employ the Extended Kalman Filter (EKF) for error state prediction and covariance propagation, and the Sigma Point Kalman Filter (SPKF) for robust measurement updating in the presence of high nonlinearities. The outdoor and indoor experiments show that the combination of point and line features improves the estimation accuracy and robustness compared to the algorithm using point features alone.

## 1. Introduction

Reliable navigation in dynamic and unknown environments is a key requirement for many applications, particularly for autonomous ground, underwater and air vehicles. The most common sensor modality used to tackle this problem is the Inertial Measurement Unit (IMU). However, inertial navigation systems (INS) are proved to drift over time due to error accumulation [1]. In the last decades, the topic of vision-aided inertial navigation has received considerable attention in the research community, thanks to some important advantages [2,3,4,5,6,7,8,9]. Firstly, the integrated system can operate in environments where GPS is unreliable or unavailable. Secondly, the complementary frequency responses and noise characteristics of vision and inertial sensors address the respective limitations and deficiencies [10]. In particular, fast and highly dynamic motions can be precisely tracked by an IMU in a short time, and thus the problem of scale ambiguity and large latency in vision can be settled to a certain extent. On the other hand, the low-frequency drift in the inertial measurements can be significantly controlled by visual observations. Furthermore, both cameras and IMUs are low cost, light-weight and low power-consumption devices, which make them ideal for many payload-constrained platforms. Corke [10] has presented a comprehensive introduction of these two sensory modalities from a biological and an engineering perspective.

The simplest fusion scheme for a vision-aided inertial navigation system (VINS) uses separate INS and visual blocks, and fuses information in a loosely-coupled approach [10]. For instance, some methods fuse the inertial navigation solution with the relative pose estimation between consecutive image measurements [11,12,13,14]. Tightly-coupled methods in contrast process the raw information of both sensors in a single estimator, thus all the correlations between them are considered, leading to higher accuracy [15,16]. The most common tightly-coupled scheme augments the 3D feature positions in the filter state, and concurrently estimates the motion and structure [2]. However, this method suffers from high computational complexity, as the dimension of the state vector increases with the number of the observed features. To address this problem, Mourikis [15] proposed an EKF-based algorithm which maintains a sliding window of poses in the filter state, and make use of the tracked features to impose constraints on these poses. The shortcomings of this approach are twofold: (1) the space complexity is high, because it needs to store all the tracked features; (2) it requires a reconstruction of the 3D position of the tracked feature points, which are not necessary in navigation tasks. To overcome these shortcomings, Hu [9] developed a sliding window odometry using the monocular camera geometry constraints among three images as measurements, resulting in a tradeoff between accuracy and computational cost.

While the vision-aided inertial navigation has been extensively studied, and a considerable amount of work has also been dedicated to processing visual observations of point features [2,4,5,7], on the contrary, much less work has been aimed at exploring line features. In fact, line primitives and point primitives provide complementary information about the image [17]. There are many scenes (e.g., wall corners, stairwell edges, *etc.*) where the point primitive matches are unreliable while the line primitives are well matched, due to multi-pixel support [6].

On the other hand, points are crucial as they give more information than lines. For instance, there are no pose constraints imposed by line correspondences from two views, while there are well-known epipolar geometry constraints for point correspondences from two views [18].

In this paper, we propose a method that combines point and line features for navigation aiding in a simple and unified framework. Our algorithm can deal with any mixed combination of point and line correspondences utilizing trifocal geometry across two stereo views. In the implementation, the inertial sensors are tightly-coupled within feature tracking to improve the robustness and tracking speed. Meanwhile, the drifts of inertial sensors are greatly reduced by using the constraints imposed in the tracked features. Leveraging both of the complementary characteristics of the inertial and visual sensors and the complementary characteristics between point and line features, the proposed algorithm demonstrates improved performance and robustness.

The remainder of this paper is organized as follows: we describe the mathematical model of the VINS in Section 2, and then develop our estimator in Section 3. Experimental results are given in Section 4. Finally, Section 5 contains some conclusions and suggests several directions for future work.

## 2. Mathematical Formulation

### 2.1. Notations and Convention

We denote scalars in italic lower case letters (e.g., a), denote vectors in lower case letters with boldface non-italic (e.g., p), and denote matrices in upper case letters with bold font (e.g., R). If a vector or matrix describes the relative pose of one reference frame with respect to another, we combine subscript letters to designate the frames, e.g., $p_{WI}$ represents the translation vector from the origin of the frame {W} to the origin of the frame {I}, and $R_{WI}$ represents the direction cosine matrix of frame {I} in the reference frame {W}. The six degrees of freedom transform between two reference frames can be represented as a translation followed by a rotation:

(1) $$t^{W}=p_{WI}^{W}+R_{WI}t^{I}$$

In the remaining Sections, unit quaternions are also used to describe the relative orientation of two reference frames, e.g., ${\bar{q}}_{WI}$ represents the orientation of frame {I} in frame {W}.

Finally, to represent projective geometry, it is simpler and more symmetric to introduce homogeneous coordinates, which provides a scale invariant representation for point and line in the Euclidean plane. In this paper, vectors in homogeneous coordinate form are expressed by an underline, e.g., $\underline{m}={\left(\begin{array}{ccc} u & v & w \end{array}\right)}^{T}$ represents the point $m={\left(\begin{array}{cc} u\prime & v\prime \end{array}\right)}^{T}$ in the Euclidean plane, with u′=u/w,v′=v/w,w≠0.

### 2.2. System Model

The evolving IMU state is described by the vector:

(2) $$x_{IMU}\left(t\right)={\left[\begin{array}{ccccc} {\left(p_{WI}^{W}\right)}^{T} & {\left({\bar{q}}_{WI}\right)}^{T} & {\left(v_{WI}^{W}\right)}^{T} & {\left(b_{g}\left(t\right)\right)}^{T} & {\left(b_{a}\left(t\right)\right)}^{T} \end{array}\right]}^{T}$$

where $p_{WI}^{W}\left(t\right)$ denotes the position of IMU in the world frame {W}; ${\bar{q}}_{WI}\left(t\right)$ is the unit quaternion of the IMU frame {I} in the world frame; $v_{WI}^{W}$ is the linear velocity of the IMU in the world frame; $b_{g}\left(t\right)$ and $b_{a}\left(t\right)$ are the IMU gyroscope and accelerometer biases, respectively.

In this work, we model the biases $b_{g}\left(t\right)$ and $b_{a}\left(t\right)$ as a Gaussian random walk process, driven by the white, zero-mean noise vectors $n_{gw}$ and $n_{aw}$, with covariance matrices $Q_{gw}$ and $Q_{aw}$ respectively. The time evolution of the IMU state is given by the following equation [2]:

(3) $${\dot{p}}_{WI}^{W}=v_{WI}^{W},\;{\dot{\bar{q}}}_{WI}=\frac{1}{2}\Omega \left(\omega_{WI}^{I}\right){\bar{q}}_{WI},\;{\dot{v}}_{WI}^{W}=a_{WI}^{W},\;{\dot{b}}_{g}=n_{gw},\;{\dot{b}}_{a}=n_{aw}$$

where $\Omega \left(\omega_{WI}^{I}\right)$ is the quaternion multiplication matrix:

(4) $$\Omega \left(\omega_{WI}^{I}\right)=\left[\begin{array}{cc} 0 & -{\left(\omega_{WI}^{I}\right)}^{T}\\ \omega_{WI}^{I} & -\left[\omega_{WI}^{I}\times \right] \end{array}\right]$$

which relates the time rate of change of the unit quaternion to the angular velocity; $\omega_{WI}^{I}$ is the angular velocity of the IMU with respect to the world frame, and $a_{WI}^{W}$ is the acceleration of the IMU with respected to the world frame expressed in the world frame. The measured angular velocity and linear acceleration from are:

(5) $$\omega_{m}=\omega_{WI}^{I}+b_{g}+n_{g}$$

(6) $$a_{m}=R^{T}\left({\bar{q}}_{WI}\right)\left(a_{WI}^{W}-g^{W}\right)+b_{a}+n_{a}$$

where $R\left({\bar{q}}_{WI}\right)$ is the direction cosine matrix corresponding to the unit quaternion ${\bar{q}}_{WI}$, $n_{g}$ and $n_{a}$ are measurements noises of gyroscope and accelerometer, which are assumed to be zero-mean Gaussian noise with covariance matrices $Q_{g}$ and $Q_{a}$, respectively. Note that we do not consider the Earth’s rotation rate in the gyroscope measurement, because it is small enough relative to the noise and bias of the low-cost gyroscope.

### 2.3. Measurement Model

#### 2.3.1. Camera Model

In this Section, we consider the standard perspective camera model, which is commonly used in the computer vision applications. Let K denote the intrinsic camera parameters matrix which can be obtained by calibrating. A mapping between the 3D homogeneous point $\underline{M}={\left[\begin{array}{cccc} M_{1} & M_{2} & M_{3} & M_{4} \end{array}\right]}^{T}$ in space and the homogeneous image pixel coordinates $\underline{m}={\left[\begin{array}{ccc} u & v & 1 \end{array}\right]}^{T}$ can be given by:

(7) $$\underline{m}\propto K\cdot \left[R|t\right]\underline{M}=P\underline{M}$$

where ∝ means equality up to scale, and P=K⋅[R|t] is 3 × 4 camera matrix, with **R** and t representing pose of the camera with respect to the world reference frame. Similarly, a mapping between a 3-space line represented as a Plücker matrix L and the homogenous image line l is given by [18]:

(8) $$\left[l\times \right]=PLP^{T}$$

#### 2.3.2. Review of the Trifocal Tensor

A trifocal tensor is a 3 × 3 × 3 array of numbers that describes the geometric relations among three views. It depends only on the relative motion between the different views and is independent of scene structures. Assuming that the camera matrices of three views are $P_{1}=\left[I|0\right],$ $P_{2}=\left[A|a_{4}\right],$ $P_{3}=\left[B|b_{4}\right]$, the entries of the trifocal tensor can be derived accordingly using the standard matrix-vector notation [18]:

(9) $$T_{i}=a_{i}b_{4}^{T}-a_{4}b_{i}^{T}$$

where $a_{i}$ and $b_{i}$ denote the i-th column of the camera matrices $P_{2}$ and $P_{3}$, respectively.

**Figure 1:**
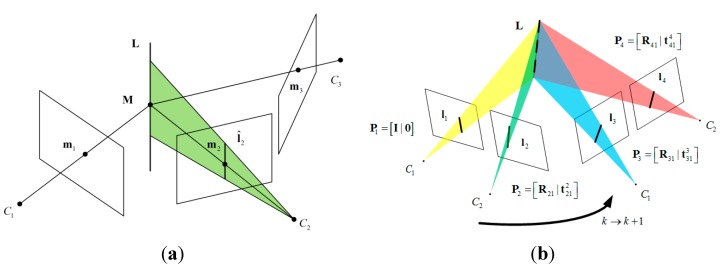
(**a**) The point-line-point correspondence among three views; (**b**) Stereo geometry for two views and line-line-line configuration.

Once the trifocal tensor is computed, we can use of it to map a pair of matched points ${\underline{m}}_{1}↔{\underline{m}}_{2}$ in the first and second views into the third view, using the homography between the first view and the third view induced by a line in the second image [18]. As shown in Figure 1a, a line in second view defines a plane in space, and this plane induces a homography between the first view and third view. As recommended by Hartley [18], the line ${\hat{l}}_{2}$ is chosen as the line perpendicular to the epipolar line. The transfer procedure is summarized as follows [18]:

(1) Compute the epipolar line $l_{e}=F_{21}{\underline{m}}_{1}$, where $F_{21}$ is the fundamental matrix between the first and second views.
(2) Compute the line ${\hat{l}}_{2}$ which passes through ${\underline{m}}_{2}$ and is perpendicular to $l_{e}$. If $l_{e}={\left[\begin{array}{ccc} l_{e1} & l_{e2} & l_{e3} \end{array}\right]}^{T}$ and ${\underline{m}}_{2}={\left[\begin{array}{ccc} m_{21} & m_{22} & 1 \end{array}\right]}^{T}$, then ${\hat{l}}_{2}={\left[\begin{array}{ccc} l_{e2} & -l_{e1} & -m_{21}l_{e2}+m_{22}l_{e1} \end{array}\right]}^{T}$.
(3) The transferred point is ${\hat{\underline{m}}}_{3}=\left(\sum_{i}m_{1i}T_{i}^{T}\right){\hat{l}}_{2}.$

Similarly, it is possible to transfer a pair of matched lines $l_{2}↔l_{3}$ in the second and third views into the first view according to the line transfer equation [18]:

(10) $${\hat{l}}_{1i}=l_{2}^{T}T_{i}l_{3}$$

#### 2.3.3. Stereo Vision Measurement Model via Trifocal Geometry

In this Section, we exploit the trifocal geometry of stereo vision to deduce the measurement model. We depict the stereo camera configuration of two consecutive frames in Figure 1b. For the sake of clarity, we only provide the geometrical relations of lines. The camera matrices of the stereo image pair at the previous time step can be represented in canonical form as:

(11) $$P_{1}=\left[I|0\right],P_{2}=\left[R_{21}|t_{21}^{2}\right]$$

where $R_{21}=R_{0}$ and $t_{21}^{2}=t_{0}$ encode the rigid transform of the rig which are known after calibration. The camera matrices of the successive stereo image pairs are defined as:

(12) $$P_{3}=\left[R_{31}|t_{31}^{3}\right],P_{4}=\left[R_{41}|t_{41}^{4}\right]$$

For simplicity, we assume that the IMU frame of reference coincides with the camera frame of reference. Thus, the terms in Equation (12) can be expressed as follows:

(13) $$R_{31}=R_{WI}^{T}R_{WI_{1}}$$

(14) $$t_{31}^{3}=R_{WI}^{T}\left(p_{WI_{1}}^{W}-p_{WI}^{W}\right)$$

(15) $$R_{41}=R_{43}R_{31}=R_{0}R_{WI}^{T}R_{WI_{1}}$$

(16) $$t_{41}^{4}=t_{43}^{4}+R_{43}t_{31}^{3}=t_{0}+R_{0}R_{WI}^{T}\left(p_{WI_{1}}^{W}-p_{WI}^{W}\right)$$

where $R_{WI_{1}}$ and $p_{WI_{1}}^{W}$ are the pose of IMU corresponding to the last time the image pair captured.

Two trifocal tensors, $T_{L}=\left\{T_{i}^{L}\right\}$ relating the previous image pair to the current left image and $T_{R}=\left\{T_{i}^{R}\right\}$ relating to the current right image can be determined according to Equation (9) using the camera matrices Equations (11) and (12):

(17) $$T_{L}=T\left(P_{1},P_{2},P_{3}\right)=T\left(R_{0},t_{0},R_{WI},p_{WI}^{W},R_{WI_{1}},p_{WI_{1}}^{W}\right)$$

(18) $$T_{L}=T\left(P_{1},P_{2},P_{4}\right)=T\left(R_{0},t_{0},R_{WI},p_{WI}^{W},R_{WI_{1}},p_{WI_{1}}^{W}\right)$$

From the corresponding point set $\left\{m_{1}↔m_{2}↔m_{3}↔m_{4}\right\}$ and the point transfer relations among the triplets, the following non-linear functions can be defined:

(19) $$h_{1}\left(T_{L}\left(R_{0},t_{0},R_{WI},p_{WI}^{W},R_{WI_{1}},p_{WI_{1}}^{W}\right),m_{1},m_{2},m_{3}\right)=0_{2\times 1}$$

(20) $$h_{1}\left(T_{R}\left(R_{0},t_{0},R_{WI},p_{WI}^{W},R_{WI_{1}},p_{WI_{1}}^{W}\right),m_{1},m_{2},m_{4}\right)=0_{2\times 1}$$

where $h_{1}\left(\cdot \right)$ denotes the pixel differences between the transferred point and the measured point.

For line measurements, we also need a formulation to compare the transferred lines with the measured lines. Because of the aperture problem [19], only the measurement components which are orthogonal to the transferred line can be used for correction. In [3,17], the line-point is chosen as observation, which is defined as the closest point on the line segment to the image origin. Accordingly, the error function is defined as the differences between the measured and transferred line-points, which is similar to the error function of point features. However, when the lines pass through the origin, the orientation error of the lines cannot be revealed by this error function. Thus, we choose the signed distances between the endpoints of the measured line segment to the transferred line as observation. Suppose that ${\underline{s}}^{a}$ and ${\underline{s}}^{b}$ are the end points of the line segment measured in the first view. We denote the line transferred from the second and third views by $\hat{l}={\left({\hat{l}}_{1},{\hat{l}}_{2},{\hat{l}}_{3}\right)}^{T}$. The signed distances between the end points of the measured line segment and the transferred line make up the line observation function:

(21) $$d_{1}=\left[\begin{array}{c} {\underline{s}}^{a}\cdot \hat{l}/\sqrt{{\left({\hat{l}}_{1}\right)}^{2}+{\left({\hat{l}}_{2}\right)}^{2}}\\ {\underline{s}}^{b}\cdot \hat{l}/\sqrt{{\left({\hat{l}}_{1}\right)}^{2}+{\left({\hat{l}}_{2}\right)}^{2}} \end{array}\right]=0_{2\times 1}$$

Similarly, the line observation function concerning the first, second, and fourth views is defined as:

(22) $$d_{2}=\left[\begin{array}{c} {\underline{s}}^{a}\cdot \hat{l}\prime /\sqrt{{\left({\hat{l}}_{1}\prime \right)}^{2}+{\left({\hat{l}}_{2}\prime \right)}^{2}}\\ {\underline{s}}^{b}\cdot \hat{l}\prime /\sqrt{{\left({\hat{l}}_{1}\prime \right)}^{2}+{\left({\hat{l}}_{2}\prime \right)}^{2}} \end{array}\right]=0_{2\times 1}$$

where $\hat{l}\prime ={\left({\hat{l}}_{1}\prime ,{\hat{l}}_{2}\prime ,{\hat{l}}_{3}\prime \right)}^{T}$ is the line transferred from the second and fourth views.

As we process the point and line measurements in a unified manner after defining the corresponding error, we define the observation model in a single function:

(23) $$z=h\left(T_{L}\left(R_{0},t_{0},R_{WI},p_{WI}^{W},R_{WI_{1}},p_{WI_{1}}^{W}\right),T_{R}\left(R_{0},t_{0},R_{WI},p_{WI}^{W},R_{WI_{1}},p_{WI_{1}}^{W}\right),\left\{f_{1},f_{2},f_{3},f_{4}\right\}\right)=0_{4\times 1}$$

where $\left\{f_{1},f_{2},f_{3},f_{4}\right\}$ denotes the general feature correspondences among the four views, $T_{L}$ and $T_{R}$ encode the motion information between the successive stereo image pairs, and the function h(⋅) defines the observations based on the feature type.

## 3. Estimator Description

### 3.1. Structure of the State Vector

As can be seen in the previous Section, the measurement models are implicit relative-pose measurements, which relate the system state at two different time instants (*i.e.*, the current time and the previous time when image pair is captured). However, the “standard” Kalman filter formulation requires that the measurements employed for the state update be independent of any previous filter states. The problem can be addressed by augment the state vector to include a history of IMU pose when last image pair is recorded. With these state augmentations, the measurements are only related to the current state, and thus, a Kalman filter framework can be applied. The augmented nominal state is given by:

(24) $$\hat{x}={\left[\begin{array}{ccc} {\hat{x}}_{IMU}^{T} & {\left({\hat{p}}_{WI_{1}}^{W}\right)}^{T} & {\left({\hat{\bar{q}}}_{I_{1}}^{W}\right)}^{T} \end{array}\right]}^{T}$$

where ${\hat{x}}_{IMU}^{T}$ is the nominal state of IMU, which can be obtained by integrating Equation (3) without considering the noise term; ${\left({\hat{p}}_{WI_{1}}^{W}\right)}^{T}$ and ${\left({\hat{\bar{q}}}_{I_{1}}^{W}\right)}^{T}$ denotes the nominal-state pose of the IMU at time when the last image pair is recorded. The augmented error state is defined accordingly:

(25) $$\delta x={\left[\begin{array}{ccc} \delta x_{IMU} & {\left(\delta p_{WI_{1}}^{W}\right)}^{T} & {\left(\delta \theta^{I_{1}}\right)}^{T} \end{array}\right]}^{T}$$

where $\delta x_{IMU}$ is the IMU error-state defined as:

(26) $$\delta x_{IMU}={\left[\begin{array}{ccccc} {\left(\delta p_{WI}^{W}\right)}^{T} & {\left(\delta \theta^{I}\right)}^{T} & {\left(\delta v_{WI}^{W}\right)}^{T} & \delta b_{g}^{T} & \delta b_{a}^{T} \end{array}\right]}^{T}$$

The standard additive error definition is used for the position, velocity and biases, while for the orientation error $\delta \theta^{I}$, the multiplicative error definition is applied:

(27) $${\bar{q}}_{I}^{W}={\hat{\bar{q}}}_{I}^{W}⊗{\left[\begin{array}{cc} 1 & \frac{1}{2}{\left(\delta \theta^{I}\right)}^{T} \end{array}\right]}^{T}$$

where the symbol ⊗ denotes quaternion multiplication. With the above error definition, the true-state may be expressed as a suitable composition of the nominal and the error-states:

(28) $$x=\hat{x}⊕\delta x$$

where ⊕ means a generic composition.

### 3.2. Filter Propagation

The continuous-time IMU error-state model may be given as a single matrix error equation:

(29) $$\delta {\dot{x}}_{IMU}=F_{IMU}\delta x_{IMU}+G_{IMU}n_{IMU}$$

where:

(30) $$F_{IMU}=\left[\begin{array}{ccccc} 0_{3\times 3} & I_{3} & 0_{3\times 3} & 0_{3\times 3} & 0_{3\times 3}\\ 0_{3\times 3} & -\left\lfloor \left(\omega_{WI}^{I}\left(t\right)-b_{g}\left(t\right)\right)\times \right\rfloor & 0_{3\times 3} & -I_{3} & 0_{3\times 3}\\ 0_{3\times 3} & -R\left({\bar{q}}_{WI}\left(t\right)\right)\left\lfloor \left(a_{m}\left(t\right)-b_{a}\left(t\right)\right)\times \right\rfloor & 0_{3\times 3} & 0_{3\times 3} & -R\left({\bar{q}}_{WI}\left(t\right)\right)\\ 0_{3\times 3} & 0_{3\times 3} & 0_{3\times 3} & 0_{3\times 3} & 0_{3\times 3}\\ 0_{3\times 3} & 0_{3\times 3} & 0_{3\times 3} & 0_{3\times 3} & 0_{3\times 3} \end{array}\right]$$

(31) $$G_{IMU}=\left[\begin{array}{cccc} 0_{3\times 3} & 0_{3\times 3} & 0_{3\times 3} & 0_{3\times 3}\\ -I_{3} & 0_{3\times 3} & 0_{3\times 3} & 0_{3\times 3}\\ 0_{3\times 3} & -R\left({\bar{q}}_{WI}\left(t\right)\right) & 0_{3\times 3} & 0_{3\times 3}\\ 0_{3\times 3} & 0_{3\times 3} & I_{3} & 0_{3\times 3}\\ 0_{3\times 3} & 0_{3\times 3} & 0_{3\times 3} & I_{3} \end{array}\right]$$

(32) $$n_{IMU}={\left[\begin{array}{cccc} {\left(n_{g}\right)}^{T} & {\left(n_{a}\right)}^{T} & {\left(n_{gw}\right)}^{T} & {\left(n_{aw}\right)}^{T} \end{array}\right]}^{T}$$

Since the past pose is unchanged during the filter prediction step, its corresponding derivatives are zero:

(33) $${\dot{\hat{p}}}_{WI_{1}}^{W}=0,{\dot{\hat{\bar{q}}}}_{I_{1}}^{W}=0$$

(34) $$\delta {\dot{p}}_{WI_{1}}^{W}=0,\delta {\dot{\theta }}^{I_{1}}=0$$

Combining Equations (29) and (34), the continuous-time augmented error state equation is given by:

(35) $$\delta \dot{x}=F_{c}\delta x+G_{c}n_{IMU}$$

where:

(36) $$F_{c}=\left[\begin{array}{cc} F_{IMU} & 0_{15\times 6}\\ 0_{6\times 15} & 0_{6\times 6} \end{array}\right]$$

(37) $$G_{c}=\left[\begin{array}{c} G_{IMU}\\ 0_{6\times 6} \end{array}\right]$$

where $F_{IMU}$ and $G_{IMU}$ are defined in Equations (30) and (31).

Each time a new IMU measurement is received, the nominal state prediction is performed by numerical integration of the kinematic Equations (3) and (33). In order to obtain the error covariance, we compute the discrete-time state transition matrix:

(38) $$\Phi_{k}=\Phi \left(t_{k+1},t_{k}\right)=\exp\left(\int_{t_{k}}^{t_{k+1}}F_{c}\left(\tau \right)d\tau \right)$$

The elements of $\Phi_{k}$ can be computed analytically following similar derivation as [20]. The state transition matrix is slightly different from [21], because the rates of filter prediction and filter update are different in our case.

The noise covariance matrix $Q_{d}$ of the discrete-time system is evaluated by:

(39) $$Q_{d}=\int_{t_{k}}^{t_{k+1}}\Phi \left(t_{k+1},\tau \right)G_{c}Q_{c}G_{c}^{T}\Phi^{T}\left(t_{k+1},\tau \right)d\tau$$

The predicted covariance is then obtained as:

(40) $$P_{k+1|k}=\Phi_{k}P_{k|k}\Phi_{k}^{T}+Q_{d}$$

### 3.3. Measurement Update

Since the measurement model is highly nonlinear, we employ statistical linearization for measurement updating, which is generally more accurate than the first order Taylor series expansion [22]. Specifically, the Sigma Point approach is applied. First, the following sets of sigma points are selected:

(41) $$\begin{array}{l} X^{\left(0\right)}=0_{27\times 1},\;\\ X^{\left(i\right)}={\left(\sqrt{\left(n+\lambda \right)P_{k+1|k}}\right)}_{i},\;i=1,...,n,\\ X^{\left(i\right)}=-{\left(\sqrt{\left(n+\lambda \right)P_{k+1|k}}\right)}_{i},\;i\;=\;n+1,...,2n \end{array}$$

where n=21 is the dimension of the state, the parameter $\lambda =\alpha^{2}\left(n+\kappa \right)-n$ with tuning parameters α,κ, ${\left(\sqrt{P}\right)}_{i}$ indicates the ith column of the matrix square-root of the covariance matrix P. We define the following weights for the sigma points:

(42) $$\begin{array}{l} W_{m}^{\left(0\right)}=\frac{\lambda }{\lambda +n},\\ W_{c}^{\left(0\right)}=\frac{\lambda }{\lambda +n}+\left(1-\alpha^{2}+\beta \right),\\ W_{m}^{\left(i\right)}=W_{c}^{\left(i\right)}=\frac{1}{2\left(\lambda +n\right)},\;i=1,2,...,2n \end{array}$$

where β is related to the higher order moments of the distribution [23] (a good starting guess is β=2 for Gaussian distribution).

The predicted measurement vector is determined by propagating individual sigma point through the nonlinear observation function h(⋅) defined in Equation (23):

(43) $$Z_{j}^{\left(i\right)}=h\left(T_{L}\left({\hat{x}}_{k+1}⊕X^{\left(i\right)}\right),T_{R}\left({\hat{x}}_{k+1}⊕X^{\left(i\right)}\right),{\left\{f_{1},f_{2},f_{3},f_{4}\right\}}_{j}\right)$$

The mean and covariance are computed as:

(44) $${\hat{Z}}_{j}=\sum_{i=0}^{i=2L}W_{m}^{\left(i\right)}Z_{j}^{\left(i\right)}$$

(45) $$P^{z_{j}z_{j}}=\sum_{i=0}^{i=2L}W_{c}^{\left(i\right)}\left[Z_{j}^{\left(i\right)}-{\hat{Z}}_{j}\right]{\left[Z_{j}^{\left(i\right)}-{\hat{Z}}_{j}\right]}^{T}$$

(46) $$P^{xz_{j}}=\sum_{i=0}^{i=2L}W_{c}^{\left(i\right)}\left[X^{\left(i\right)}-0_{27\times 1}\right]{\left[Z_{j}^{\left(i\right)}-{\hat{Z}}_{j}\right]}^{T}$$

where $P^{z_{j}z_{j}}$ and $P^{xz_{j}}$ are the predicted measurement covariance matrix and the state-measurement cross-covariance matrix, respectively.

The filter gain is given as follows:

(47) $$K_{j}=P^{xz_{j}}{\left(P^{z_{j}z_{j}}+R_{j}\right)}^{-1}$$

where $R_{j}$ is the measurement noise covariance matrix.

Then, the error state and error covariance are updated using the normal Kalman filter equation:

(48) $$\delta x_{k+1|k+1}=\delta x_{k+1|k}+K_{j}\left(0-{\hat{Z}}_{j}\right)$$

(49) $$P_{k+1|k+1}=P_{k+1|k}-K_{j}P^{z_{j}z_{j}}K_{j}^{T}$$

After measurement update, the estimated state $\delta x_{k+1|k+1}$ is then used to correct nominal state ${\hat{x}}_{k+1}$.

Finally, replace old state by current state and revise the corresponding error covariance:

(50) $${\hat{x}}_{k+1}=T_{n}{\hat{x}}_{k+1},\;\delta x_{k+1}=T_{e}\delta x_{k+1},P_{k+1|k+1}=T_{e}P_{k+1|k+1}T_{e}^{T}$$

with:

(51) $$T_{n}=\left[\begin{array}{ccc} I_{7} & 0_{7\times 9} & 0_{7\times 7}\\ 0_{9\times 7} & I_{9} & 0_{9\times 7}\\ I_{7} & 0_{7\times 9} & 0_{7\times 7} \end{array}\right],T_{e}=\left[\begin{array}{ccc} I_{6} & 0_{6\times 9} & 0_{6\times 6}\\ 0_{9\times 6} & I_{9} & 0_{9\times 6}\\ I_{6} & 0_{6\times 9} & 0_{6\times 6} \end{array}\right]$$

## 4. Experimental Results and Discussion

### 4.1. Outdoor Experiment

We evaluate the proposed method using the publicly available KITTI Vision Benchmark Suite [24], which provides several multi-sensor datasets with ground truth. The selected dataset was captured in a residential area from the experimental vehicle, equipped with a GPS/IMU localization unit with RTK correction signals (OXTS RT3003), and a stereo rig with two grayscale cameras (PointGrey Flea2). The duration is about 440 s, with a traveling distance of about 3600 m, and the average speed is about 29 km/h. All the sensors are rigidly mounted on top of the vehicle. The intrinsic parameters of the cameras and the transformation between the cameras and GPS/IMU were well calibrated. Moreover, the cameras and GPS/IMU are manually synchronized, with sampling rates of 10 Hz and 100 Hz, respectively.

The announced gyroscope and accelerometer bias specifications are 36 deg/h (1 σ) and 1 mg (1 σ), respectively. The resolution of stereo images is 1226 × 370 pixels, with 90° field view. For the position ground truth, we use the trajectory of the GPS/IMU output, with open sky localization errors less than 5 cm.

#### 4.1.1. Feature Detection, Tracking, and Outlier Rejection

For point features, the fast corner detection (FAST) algorithm [25] was used for feature extraction, and matching was carried out by normalized cross-correlation. The main advantage of the FAST detector compared to others is the better trade-off between accuracy and efficiency. In order to reduce the computational complexity and to guarantee the well distribution of the image features, we choose a subset of the matched point features by means of bucketing [26]: Divide the image into several non-overlapping rectangles, and maintain a maximal number of feature points in each rectangle.

**Figure 2:**
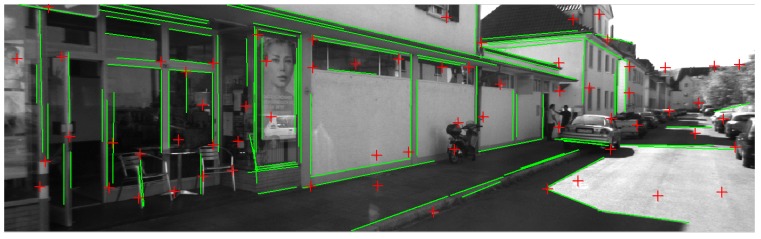
Sample image with extracted point (red) and line (green) features.

We extract lines using EDlines detector [27] in the scale space, which can give accurate results in real-time. Then we employ the method described in [28] for line matching. The lines are described local appearances by the so-called Line Band Descriptor (LBD) similar to SIFT [29] for point features, and are matched by exploiting the local appearance similarities and geometric consistencies [28]. The average execution time of line matching between views is about 56 ms with Intel Core i5 2.6 GHz processors running the non-optimized C++ code. Figure 2 shows a sample image from the dataset with extracted points and lines. As can be seen, both point and line features are rich in the selected sequence.

In order to reject mismatched features and features located on independently moving objects (e.g., the running car), we employ a chi-square test [30] for the measurement residuals. We compute the Mahalanobis distance:

(52) $$\upsilon_{j}={\left(0-{\hat{Z}}_{j}\right)}^{T}{\left(P^{z_{j}z_{j}}+R_{j}\right)}^{-1}\left(0-{\hat{Z}}_{j}\right)$$

where $\left(0-{\hat{Z}}_{j}\right)$ is the measurement residual, and $P^{z_{j}z_{j}}+R_{j}$ is the covariance of the measurement residual. The rejection threshold is usually chosen by an empirical evaluation of reliability of feature matching. We set the threshold to 12 in the experiment. The feature measurements whose residuals exceed the threshold are discarded.

#### 4.1.2. Experimental Results

In this Section, we compare the performance of our algorithm with the following methods: (1) GPS/IMU localization unit, with open sky localization errors less than 5 cm; (2) VINS using only point feature; (3) pure inertial-only navigation solution; (4) pure stereo visual odometry [31].

**Figure 3:**
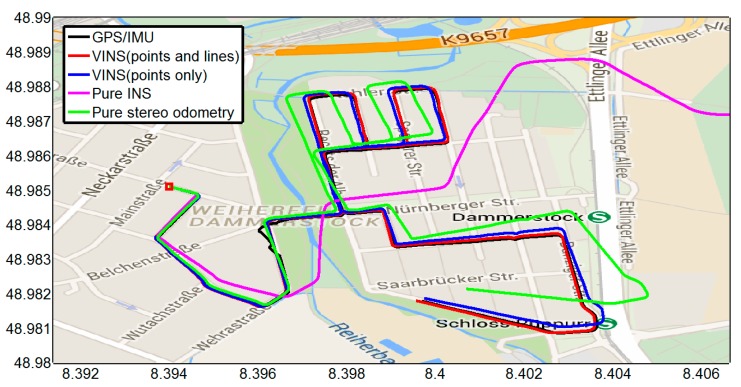
The motion trajectory plot on Google Maps. The initial position is denoted by a red square.

**Figure 4:**
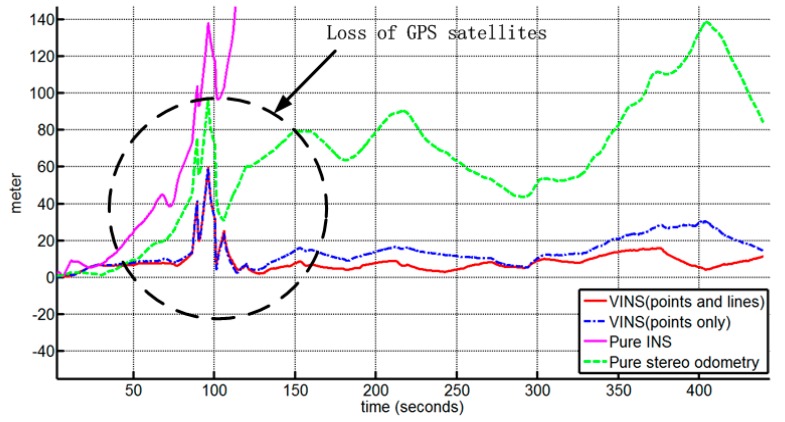
3D Position Errors of different solutions.

The trajectory estimation results of different algorithms with the ground truth data are shown in Figure 3. The corresponding 3D position errors are depicted in Figure 4. The overall root-mean-square errors (RMSE) are shown in Table 1. It can be found that the inertial-only navigation suffers from error accumulation and is not reliable for long-term operation; Secondly, the result of pure stereo visual odometry is inferior, specially where the vehicle turns, and the error grows super-linearly owing to the inherent bias in stereo visual odometry; Thirdly, the combining of inertial navigation and stereo vision with point feature alone can reduce the drift rate effectively, and the additional information from line measurements results in better performance. Note that the jumps from 80 s to 100 s are caused by ground truth errors. It also shows the advantage of the proposed method in cluttered urban environments where the GPS information is less reliable.

**Table 1:** The overall RMSE of the outdoor experiment.

Methods	Position RMSE (m)	Orientation RMSE (deg)
VINS (points and lines)	10.6338	0.8313
VINS (points only)	16.4150	0.9126
Pure INS	2149.9	2.0034
Pure stereo odometry	72.6399	8.1809

**Figure 5:**
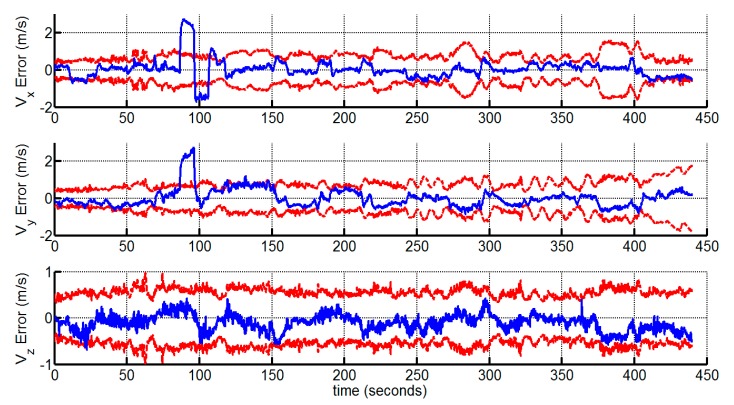
The velocity estimation errors and 3 σ bounds (the large deviations around 100th second is due to the ground truth errors).

**Figure 6:**
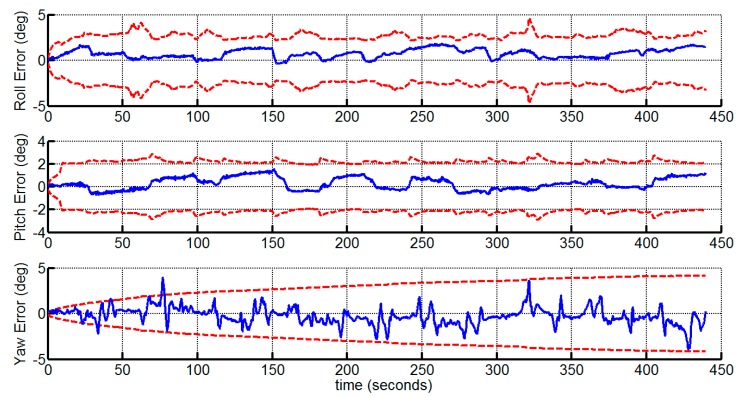
The attitude estimation errors and 3 σ bounds.

We demonstrate the velocity and attitude deviations of the proposed method with the corresponding 3 σ bounds in Figure 5 and Figure 6, which verify that the velocity and attitude estimates are consistent. Note that the standard deviations of the roll and pitch angle errors are bounded, while the standard deviation of the yaw angle error grows over time. This is consistent with the observable property of the VINS system, which indicates that the yaw angle is unobservable [8]. The yaw angle error is bounded under 5° due to the accuracy of the gyroscopes in the experiment.

**Figure 7:**
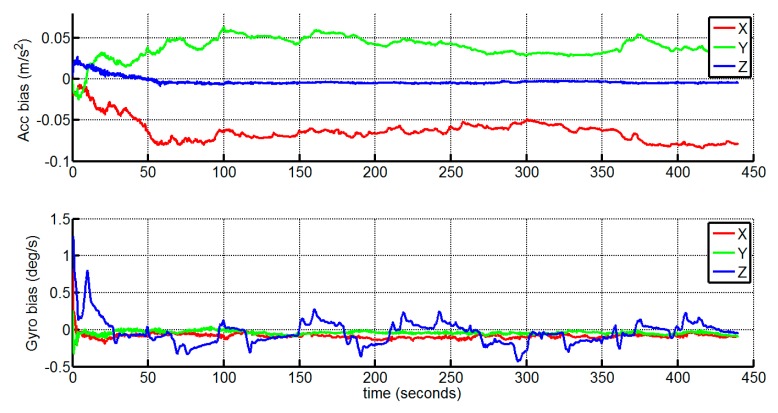
Estimated gyroscope and accelerometer bias.

Finally, the estimates of the gyroscope and accelerometer biases are depicted in Figure 7. All the estimated biases converge quickly to some reasonable ranges, meaning a practical estimation and allowing the compensation of the INS.

### 4.2. Indoor Experiment

**Figure 8:**
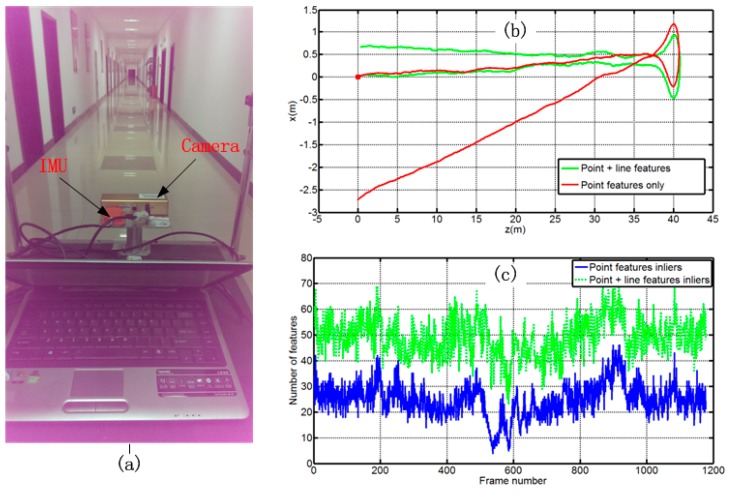
Performance in low-textured indoor environment: (**a**) Experimental setup and experimental scene; (**b**) Top view of estimated trajectories; (**c**) The number of point and line inliers used to estimate the motion.

To demonstrate the robustness of our algorithm in a textureless structured environment, we perform indoor experiments in a corridor scenario with textureless walls which lead to very few points being tracked in some frames. The test rig consists a PointGrey Bumblebee2 stereo pair, a Xsens MTi unit, and a laptop for data recording (Figure 8a). The accuracy specifications and sampling rates of the sensors are listed in Table 2. The relative pose of the IMU and the camera are well calibrated prior to the experiment using the method proposed in [32], and keep unchanged during the experiment. The actual motion of the pushcart is a move along with the corridor, and then return to the initial point. The full length of the path is about 82 m.

**Table 2:** The accuracy specifications and sampling rates of the sensors.

Sensors	Accuracies	Sampling Rates
IMU	Gyro bias stability (1 σ): 1°/s Accelerometer bias stability: 0.02 m/s^2^	100 Hz
Stereo Camera	Resolution: 640 × 480 pixels Focus length: 3.8 mm Field of view: 70° Base line: 12 cm	12 Hz

In Figure 8b, we show the bird’s eye view of the estimated trajectories. It is obvious that the combination of point and line features leads to much better performance than the use of point features alone in this scenario. The reason is that the point features are few or not well distributed over the image in some frames, leading to a bad orientation estimation. In Figure 8c, a plot of the number of inlier point and line features per frame is shown, which clearly demonstrates the superiority of combining both feature types under such circumstances.

## 5. Conclusions/Outlook

This paper presents a tightly-coupled vision-aided inertial navigation algorithm, which exploits point and line features to aid navigation in a simple and unified framework. The measurement models of the point and line features are derived, and incorporated into a single estimator. The outdoor experimental results show that the proposed algorithm performs well in cluttered urban environments. The overall RMSE of position and orientation is about 10.6 m and 0.83°, respectively, over a path of up to about 4 km in length. The indoor experiment demonstrates the better performance and robustness of combining both point and line features in textureless structured environments. The proposed approach which combines both feature types can deal with different types of environments with a slight increase in computational cost.

As part of future work, we aim to improve the proposed approach, by taking advantage of the structural regularity of man-made environments, such as Manhattan-world scenes, *i.e.*, scenes that lines should be orthogonal or parallel to each other [33]. Unlike ordinary lines, the Manhattan-world lines encode the global orientation information, which can be used to eliminate the accumulated orientation errors, and further suppress the position drifts.
